# The “ADC-after-ADC” paradigm: navigating cross-resistance and sequencing in breast cancer therapy

**DOI:** 10.3389/fonc.2026.1887974

**Published:** 2026-09-14

**Authors:** Michael Asare, Trinitas Oserefuamen Okobi, Susan Karass, Joseph Tobechukwu Okobi, Evelyn Taiwo

**Affiliations:** 1 Department of Medicine, BronxCare Health System, New York, NY, United States; 2 Division of Hematology and Medical Oncology, NewYork-Presbyterian Brooklyn Methodist Hospital, New York, NY, United States

**Keywords:** antibody-drug conjugates, circulating tumor DNA, cross-resistance, HER2-low, metastatic breast cancer, payload switching, resistance mechanisms, therapeutic sequencing

## Abstract

Antibody–drug conjugates (ADCs) are entering earlier lines of breast cancer therapy, but evidence guiding treatment after a prior ADC remains limited. We performed a structured narrative synthesis of prospective post–prior-ADC studies, peer-reviewed retrospective cohorts, human translational analyses, functional preclinical studies, regulatory documents, and trial registries through July 23, 2026. No completed trial has randomized clinically comparable patients between alternative ADC sequence orders. DESTINY-Breast02 demonstrates trastuzumab deruxtecan activity after trastuzumab emtansine, whereas DESTINY-Breast03 is a head-to-head construct comparison and not a sequential-treatment study. Retrospective cohorts show that a second ADC can retain activity, but usually report shorter progression-free survival, real-world progression-free survival, or time to treatment failure than with the first ADC. These findings cannot isolate biological cross-resistance from later treatment line, disease evolution, intervening therapy, selection, changing fitness, toxicity, and non-equivalent endpoints. Longitudinal human studies support selected target-level and payload-level mechanisms, including decreased HER2 expression and acquired *TOP1* alterations; generalized trafficking failure, efflux-mediated cross-resistance, and clinical rescue through linker or payload switching remain predominantly preclinical or hypothesis-generating. Sequential decisions should therefore be anchored to subtype-specific prospective evidence and current indications, then refined by the reason the prior ADC stopped, current target eligibility, central nervous system disease, prior toxicity, organ and marrow reserve, non-ADC alternatives, access, and patient goals. Current evidence supports individualized decision-making, not a validated universal sequence.

## 1 Introduction

Antibody–drug conjugates (ADCs) combine an antigen-directed antibody, a linker, and a cytotoxic payload. Activity depends on the complete construct and its context: target density and distribution, binding and internalization, trafficking and payload release, payload sensitivity, bystander exposure, and host tolerance (1). As ADCs move into earlier metastatic settings, treatment after a prior ADC is becoming a routine clinical problem. Recent US indications for trastuzumab deruxtecan (T-DXd), sacituzumab govitecan (SG), and datopotamab deruxtecan (Dato-DXd) enlarge the population likely to face this decision, but separate approvals do not establish an optimal order (2–4).

We use cross-resistance narrowly: reduced sensitivity to ADC2 that is mechanistically attributable to resistance selected by ADC1. Shorter progression-free survival (PFS), real-world PFS (rwPFS), duration of response, or time to treatment failure (TTF) with ADC2 does not itself prove cross-resistance. ADC2 is administered later, after additional disease evolution and often intervening treatment, to patients selected for survival and fitness; organ reserve, performance status, dose intensity, and ascertainment may also change. Conversely, ADC2 activity in some patients does not exclude shared resistance in other clones.

This review separates direct clinical, retrospective, human translational, preclinical, and hypothesis-level evidence. It asks which ADC components and resistance liabilities are shared, what sequential outcomes have actually been observed, and how subtype, target eligibility, central nervous system (CNS) disease, toxicity, alternatives, access, and patient goals should inform decisions while prospective order trials are pending.

## 2 Methods

We conducted a structured narrative literature search and evidence synthesis rather than a systematic review. PubMed/MEDLINE was searched from database inception through July 23, 2026, using combinations of terms for breast cancer; antibody–drug conjugates and individual agents; treatment sequencing; resistance and cross-resistance; ADC targets, linkers, payloads, trafficking, biomarkers, and toxicity. The search process began on December 17, 2025, and was updated through July 23, 2026. FDA documents, ClinicalTrials.gov records, and reference lists of eligible primary studies were also reviewed. A formal screening log and study-flow count were not maintained.

Eligible evidence involved adults with breast cancer and addressed at least one of the following: prospective outcomes after exposure to a specified prior ADC; peer-reviewed retrospective studies of sequential ADC therapy; paired or post-treatment human analyses of resistance mechanisms; functional preclinical evaluation of proposed resistance mechanisms; ADC construct characteristics; treatment-related toxicity; or regulatory and clinical-trial information. Full peer-reviewed primary publications were prioritized over narrative reviews, conference abstracts, and other secondary sources. Conference-only reports were used only when no full publication was available and were not used as the principal support for definitive clinical-outcome claims. Non-breast cancer studies were considered only when directly relevant to ADC pharmacology or mechanism and were identified as indirect evidence.

When multiple publications described the same trial or data source, the most mature peer-reviewed analysis was prioritized. Updated analyses were retained when they represented distinct data cutoffs or reported additional clinically relevant endpoints. Overlapping reports were identified and were not interpreted as independent replication. Numerical and clinically consequential claims were checked against accessible full-text primary publications, prescribing information, regulatory documents, or trial-registry records. Findings available only in abstracts were not interpreted beyond the populations, endpoints, and results reported by the source.

Evidence was classified as prospective clinical, retrospective clinical, human translational, preclinical, or hypothesis/expert interpretation. Prospective efficacy trials that allowed or required previous treatment with a specified ADC were distinguished from randomized trials conducted in ADC-naïve populations and from retrospective ADC-after-ADC cohorts. PFS, real-world PFS, TTF, ORR, real-world response, duration of response, and OS were treated as distinct outcomes and were not used interchangeably. Differences in breast cancer subtype, treatment line, prior ADC exposure, intervening therapy, performance status, follow-up duration, and endpoint definition were considered when interpreting studies. Because of substantial clinical and methodological heterogeneity, quantitative pooling was not performed.

This review was not conducted according to a PRISMA protocol and did not use duplicate independent screening, a formal exclusion log, or a validated risk-of-bias instrument. Accordingly, the search should not be considered exhaustive, and the possibility of study-selection and publication bias was considered when calibrating the conclusions.

## 3 Contemporary ADC landscape

The relevant pharmacologic unit is the complete ADC, not target or payload class alone (**Table 1**). T-DM1 links trastuzumab through noncleavable maleimidomethyl cyclohexane-1-carboxylate (MCC) linker to the DM1 microtubule inhibitor; its charged catabolite has limited membrane permeability. T-DXd couples trastuzumab to membrane-permeable, exatecan-derived DXd through a cleavable tetrapeptide linker. SG targets TROP2 and releases SN-38 through the hydrolysable CL2A linker, whereas TROP2-directed Dato-DXd uses a tetrapeptide-DXd platform (5–8). SG and Dato-DXd therefore share TROP2 targeting and TOP1 payload class but not antibody, linker-release chemistry, active molecule, drug-to-antibody ratio, pharmacokinetics, or toxicity.

**Table 1:** ADC architecture, toxicity, and dated US status relevant to sequencing.

ADC	Target	Linker / approximate DAR	Payload class and released molecule	Bystander evidence	Sequencing-relevant toxicity	US breast cancer status at July 23, 2026
T-DM1	HER2	Noncleavable MCC; ~3.5 (6, 25)	DM1 microtubule inhibitor; poorly membrane-permeable charged catabolite	Limited in preclinical systems	Thrombocytopenia, hepatotoxicity, neuropathy, cardiac dysfunction	Approved in defined HER2-positive early and metastatic settings
T-DXd	HER2	Cleavable tetrapeptide; ~8 (4, 7)	DXd, exatecan-derived TOP1 inhibitor; membrane permeable	Demonstrated preclinically; no standardized clinical rank	ILD/pneumonitis, nausea, cytopenias, cardiac dysfunction	Approved in defined HER2-positive, HER2-low, and HER2-ultralow settings; first-line HER2-positive with pertuzumab
SG	TROP2	Hydrolysable CL2A; ~7–8 (3, 5)	SN-38 TOP1 inhibitor; membrane permeable	Preclinical; intracellular and extracellular release may contribute	Neutropenia, diarrhea, nausea, fatigue	Approved in TNBC, including first-line indications by PD-1/PD-L1 eligibility, and later-line HR-positive/HER2-negative disease
Dato-DXd	TROP2	Cleavable tetrapeptide; ~4 (2, 8)	DXd TOP1 inhibitor; membrane permeable	Demonstrated preclinically	Stomatitis, ocular toxicity, ILD/pneumonitis, nausea	Approved in previously treated HR-positive/HER2-negative disease and TNBC when PD-1/PD-L1 therapy is unsuitable
Patritumab deruxtecan	HER3	Cleavable tetrapeptide; ~8 (40)	DXd TOP1 inhibitor; membrane permeable	Demonstrated preclinically	Cytopenias, nausea, fatigue, ILD/pneumonitis	Investigational; not FDA-approved for breast cancer
Disitamab vedotin	HER2	Protease-cleavable valine-citrulline; reported ~4 (41)	MMAE microtubule inhibitor; membrane permeable	Demonstrated preclinically	Neuropathy, neutropenia, transaminase elevation	Investigational in the United States for breast cancer

DAR values are approximate population averages. Bystander effects derive mainly from preclinical systems and should not be ranked clinically. Status was verified against FDA sources through July 23, 2026. DAR, drug-to-antibody ratio; Dato-DXd, datopotamab deruxtecan; HER2, human epidermal growth factor receptor 2; ILD, interstitial lung disease; MCC, maleimidomethyl cyclohexane-1-carboxylate; MMAE, monomethyl auristatin E; SG, sacituzumab govitecan; T-DM1, trastuzumab emtansine; T-DXd, trastuzumab deruxtecan; TNBC, triple-negative breast cancer; TOP1, topoisomerase I; TROP2, trophoblast cell-surface antigen 2.

Membrane-permeable payloads can produce bystander killing in experimental systems, but no standardized clinical assay permits cross-agent ranking. Structural differences motivate resistance hypotheses; they do not establish that a target, linker, or payload switch restores clinical sensitivity. Current US indications and label-defined populations are dated to July 23, 2026 in **Table 1**. Investigational agents are labeled as such and are not presented as available standards.

## 4 Clinical evidence for sequential ADC therapy

### 4.1 Prospective evidence after a prior ADC remains indirect

Within the PubMed/MEDLINE search, FDA documents, and ClinicalTrials.gov records reviewed through July 23, 2026, no completed randomized trial with reported results directly compared alternative ADC sequences in clinically comparable patients. Prospective ADC-sequencing studies identified in ClinicalTrials.gov were ongoing, terminated without posted results, or had no posted results as of that cutoff.

DESTINY-Breast02 randomized 608 patients with HER2-positive advanced breast cancer previously treated with T-DM1 to T-DXd or physician’s choice of capecitabine plus trastuzumab or lapatinib. Median PFS was 17.8 versus 6.9 months (hazard ratio [HR], 0.36; 95% CI, 0.28–0.45) (9). This establishes T-DXd activity in a post–T-DM1 population; it does not compare reverse order, reproduce each patient’s T-DM1 outcome within the protocol, or isolate payload switching as the cause of benefit.

DESTINY-Breast03 instead randomized participants previously treated with trastuzumab and a taxane to T-DXd or T-DM1. The primary PFS HR was 0.28 (95% CI, 0.22–0.37); the updated HR was 0.33 (95% CI, 0.26–0.43) at 28.4 months’ median follow-up (10, 11). These estimates reflect different cutoffs of the same head-to-head trial. Because participants received one randomized ADC, the study is not ADC-after-ADC evidence and cannot demonstrate rescue after T-DM1 resistance.

### 4.2 Retrospective sequencing evidence

Peer-reviewed cohorts show heterogeneous residual ADC2 activity but do not establish causality (**Table 2**). In ADC-Low, 179 patients with HER2-low metastatic breast cancer received both SG and T-DXd. Median PFS2 was 2.7 months overall, 3.1 months with T-DXd second, and 2.2 months with SG second; 54.4% had primary resistance to ADC2. Sequence was strongly associated with hormone-receptor subtype, and treatment line, intervening therapy, and eligibility for ADC2 limit order comparisons (12).

**Table 2:** Evidence for ADC treatment after prior ADC exposure.

Evidence / study	Population and sequence question	Endpoint retained as reported	Key result	Permissible inference	Principal limitations
Prospective randomized, post–prior-ADC: DESTINY-Breast02 (9)	n = 608; HER2-positive; prior T-DM1; T-DXd v capecitabine + trastuzumab/lapatinib	BICR PFS	17.8 v 6.9 months; HR 0.36 (95% CI, 0.28–0.45)	T-DXd is active after prior T-DM1	Not an order comparison; no reverse sequence; cannot isolate payload switching
Retrospective: ADC-Low (12)	n = 179; HER2-low; SG and T-DXd in either order	PFS2; primary resistance	PFS2 2.7 months overall; 3.1 with T-DXd second, 2.2 with SG second; primary resistance 54.4%	ADC2 activity is limited on average but not absent	Subtype and sequence linked; later-line and selection bias; heterogeneous intervening therapy
Retrospective: Huppert et al. (13)	n = 84; HER2-low; five US centers; SG→T-DXd or T-DXd→SG	TTF; investigator-assessed real-world response	ADC2 TTF shorter in principal groups; 19/84 (22.6%) had longer TTF with ADC2	A subset had longer TTF with ADC2 than ADC1; however, this does not establish clinical benefit.	TTF includes toxicity; non-RECIST response; small reverse-order TNBC group; frequent intervening therapy
Retrospective: Chen et al. (16)	n = 79; 64 HER2-positive, 15 HER2-low; multiple ADC constructs	PFS2; ORR	PFS2 similar by payload category; ORR differed	Residual activity across multiple construct sequences	Small, heterogeneous, nonrandom groups; target/construct/line confounding
Retrospective: Peng et al. (15)	n = 111; 83 HER2-positive, 28 HER2-low; multiple ADC constructs	PFS2	HER2-positive subgroup: 6.8 v 2.7 months for different- v similar-payload-class ADC2	Payload diversification is a testable clinical hypothesis	Nonrandom; payload covaries with target, construct, availability, line, and selection
Retrospective EHR: Tarantino et al. (14)	T-DXd cohort; TNBC subgroup with prior SG (n = 58) v no prior SG (n = 61)	rwPFS; OS	Adjusted rwPFS 3.4 v 5.7 months; OS 9.0 v 14.5 months	Prior SG is associated with shorter T-DXd outcomes	Adjusted only for prior lines in reported model; nonrandom exposure; residual confounding; cannot prove cross-resistance
Retrospective EHR: Tarantino et al. (17)	n = 793 receiving treatment after T-DXd; all subtypes	rwPFS; OS	Outcomes varied by subtype and regimen; SG immediately after T-DXd had relatively short rwPFS	Post–T-DXd effectiveness differs by regimen and subtype	Nonrandom treatment; confounding by indication; overlaps data source/calendar with Tarantino et al. (14)
Retrospective: Alaklabi et al. (42)	n = 115 SG-treated TNBC; n = 26 subsequently received T-DXd	PFS	Median PFS with T-DXd after SG 7.0 months (95% CI, 4.6–10.1)	T-DXd showed post-SG activity but did not establish sequencing benefit or absence of cross-resistance.	No matched comparator; small, selected subgroup; historical HER2-low eligibility in some patients.

DESTINY-Breast03 is intentionally excluded because it randomized T-DXd versus T-DM1 rather than protocol-defined sequential use. Non-equivalent endpoints are not pooled. BICR, blinded independent central review; EHR, electronic health record; HR, hazard ratio; ORR, objective response rate; OS, overall survival; PFS, progression-free survival; rwPFS, real-world PFS; SG, sacituzumab govitecan; T-DM1, trastuzumab emtansine; T-DXd, trastuzumab deruxtecan; TTF, time to treatment failure.

Huppert et al. evaluated 84 patients with HER2-low disease at five US centers. TTF—not PFS—was shorter with ADC2 across the principal sequence/subtype groups, although 19 of 84 patients (22.6%) had longer TTF with ADC2. Intervening therapy was frequent, and TTF included discontinuation for toxicity or other causes (13). In a nationwide electronic-health-record analysis of TNBC treated with T-DXd, prior SG exposure (n = 58) was associated with shorter rwPFS than no prior SG (n = 61; 3.4 v 5.7 months) after adjustment for prior treatment lines. Residual confounding, nonrandom exposure, and different treatment histories prevent attribution to cross-resistance (14).

In Peng et al., 111 patients—83 with HER2-positive and 28 with HER2-low disease—received sequential ADCs. Within the HER2-positive subgroup, a different-payload-class ADC2 was associated with median PFS2 of 6.8 versus 2.7 months with a similar-payload-class ADC2. Payload class covaried with construct, target, availability, treatment line, and selection; the association does not isolate a payload effect (15). Chen et al. reported a separate 79-patient cohort (64 HER2-positive, 15 HER2-low) in which PFS2 was similar by payload-category comparison, despite different response proportions (16). The studies should not be conflated.

A second nationwide electronic-health-record analysis described 793 patients receiving treatment after T-DXd; outcomes varied by subtype and regimen, and SG immediately after T-DXd was associated with relatively short rwPFS (17). This report and the prior-SG T-DXd analysis arose from the same database and overlapping calendar periods, so they are complementary analyses rather than independent confirmation. Collectively, current cohorts support two bounded conclusions: ADC2 can remain active in some patients, and average ADC2 time-to-event outcomes are often shorter in heavily pretreated populations. They do not identify a universally superior order or prove biological cross-resistance.

### 4.3 Endpoint and comparison fragility

PFS, rwPFS, TTF, ORR, real-world response, and duration of response answer different questions. rwPFS depends on nonuniform imaging and abstraction; TTF also counts discontinuation for toxicity, preference, or access; ORR depends on the assessment method and evaluable denominator; duration of response applies only to responders. Within-patient comparison does not remove later-line disease evolution, intervening therapy, changing fitness and dose intensity, survivor selection, or different assessment schedules. Across studies, subtype, target eligibility, prior ADCs, CNS disease, treatment line, practice setting, and calendar-time access vary. These studies describe associations and should not be pooled or interpreted as causal sequence tests.

## 5 Mechanism of resistance and cross-resistance

### 5.1 Target availability and heterogeneity

Target expression is a determinant of ADC delivery but is neither binary nor static. In the prospective DAISY translational study, T-DXd activity increased with HER2 expression, and 13 of 20 evaluable tumors (65%) showed decreased HER2 expression at resistance (18). Yet T-DXd uptake remained detectable in four of six resistant HER2-overexpressing tumors assessed soon after dosing, arguing against universal loss of delivery as the dominant mechanism. These small paired-biopsy analyses provide human translational evidence, not a validated rule that every tumor should switch targets after progression.

Clinical resistance to SG can also involve parallel target and payload alterations. In a deeply studied patient with TNBC, Coates and colleagues identified polyclonal acquired resistance involving *TACSTD2* alterations that impaired TROP2 membrane localization and *TOP1* alterations affecting payload sensitivity (19). This case demonstrates mechanistic heterogeneity within one patient; it cannot estimate population frequency. In ASCENT biomarker analyses, SG benefit was observed across assessed TROP2-expression groups, although the TROP2-low subgroup was small (20). Consequently, routine TROP2 IHC is not a validated binary selection test for choosing between available TROP2 ADCs.

Bystander killing may mitigate spatial antigen heterogeneity when a released payload is membrane permeable, but it does not make target expression irrelevant. Bystander potential depends on linker release, payload physicochemistry, tumor architecture, extracellular exposure, and sensitivity of neighboring cells. It should be described qualitatively rather than ranked as “potent” across agents without a standardized clinical assay.

### 5.2 Internalization, trafficking, and linker processing

Payload release is construct-specific rather than interchangeable. T-DM1 uses a noncleavable thioether linker and depends on HER2-mediated internalization followed by lysosomal degradation of the antibody to generate the active lysine–MCC–DM1 catabolite. Deruxtecan ADCs use protease-cleavable tetrapeptide linkers that release membrane-permeable DXd after intracellular processing. SG uses a hydrolysable CL2A linker that releases SN-38 through hydrolysis, allowing release both after internalization and within extracellular tumor compartments (21). Experimental models have implicated altered endocytosis, lysosomal acidification, protease activity, and drug release in resistance (6, 21, 22). Direct clinical evidence that a generalized trafficking defect drives cross-resistance across multiple breast cancer ADCs is limited.

The potential effect of switching constructs depends on the specific defect. A different linker could theoretically bypass resistance limited to a particular cleavage or release mechanism, whereas a different payload could address resistance at the intracellular payload target. Neither strategy can be assumed to restore activity when internalization, intracellular delivery, or lysosomal processing is impaired. Linker or architecture switching should therefore remain a mechanistic hypothesis until supported by biomarker-linked clinical evidence.

### 5.3 Payload-level resistance and the shared TOP1 class

T-DXd, Dato-DXd, HER3-DXd, and SG deliver TOP1-inhibiting payloads, but DXd and SN-38 are pharmacologically distinct. Differences in potency, membrane permeability, transporter susceptibility, linker stability, release kinetics, and payload exposure could influence resistance. A shared payload class therefore creates biologic plausibility for overlapping resistance, not proof of complete clinical cross-resistance.

In an observational human plasma-genotyping study, Abelman and colleagues identified acquired *TOP1* mutations at progression on a TOP1-directed ADC in 4 of 31 patients (12.9%), compared with 3 of 420 patients (0.7%) with metastatic breast cancer who had not received an ADC (23). Functional assays of three mutant TOP1 proteins demonstrated reduced enzymatic activity and resistance to both SN-38 and DXd. Among the small, selected group with mutation-associated sequential exposure, median treatment duration was 455 days with ADC1 and 52 days with ADC2. These findings support *TOP1* mutation as a candidate mechanism of shared-payload resistance, but selection for available post-treatment genotyping, the small number of cases, and the absence of prospective validation preclude its use as a clinical exclusion biomarker.

In the exploratory paired-biopsy analysis from DAISY, *SLX4* mutations were identified in 3 of 21 T-DXd resistance biopsies; only one was confirmed absent from the matched baseline specimen, whereas another was present at baseline and the third lacked an available baseline specimen (18). In separate breast cancer cell-line experiments, *SLX4* silencing increased resistance to DXd. No *TOP1* mutations were detected in the small DAISY resistance-biopsy set. Thus, *SLX4*-associated resistance remains a translational candidate supported by limited patient-derived observations and functional cell-model evidence.

Rampa and colleagues generated T-DXd- and SG-resistant breast cancer models in which payload-related mechanisms, including increased efflux-transporter activity, were implicated (24). In these preclinical *in vitro* and *in vivo* models, ADCs carrying non-TOP1 payloads restored antitumor activity. These findings support prospective evaluation of payload diversification but do not establish that payload switching improves outcomes in patients or should routinely determine ADC sequencing.

### 5.4 Efflux, DNA repair, microenvironment, and host context

ABC transporters can lower intracellular exposure to susceptible ADC catabolites, and DNA-damage response pathways can alter sensitivity to TOP1 inhibition (22, 24). Evidence is agent-, model-, and payload-dependent; “universal efflux resistance” is not established. Similarly, immune and stromal context may influence efficacy, but current assays do not identify a clinically actionable microenvironmental resistance state for ADC2 selection.

Host factors should not be mislabeled as tumor cross-resistance. Declining marrow reserve, hepatic dysfunction, frailty, prior ILD, dose reductions, and treatment discontinuation can shorten TTF or delivered exposure without demonstrating a resistant tumor mechanism.

## 6 Target switching versus payload switching

Target and payload switching test different hypotheses. A HER2-to-TROP2 switch could restore target engagement if reduced HER2 availability were relevant and TROP2 remained accessible, but it does not remove downstream processing or TOP1 liability. T-DXd→SG changes target, antibody, linker, and released molecule while retaining TOP1 inhibition; Dato-DXd→SG retains both TROP2 and TOP1 class while changing construct and release chemistry. Conversely, T-DXd→T-DM1 changes payload class while retaining HER2 dependence. Selected *TOP1* mutations with resistance to both DXd and SN-38 illustrate why target switching may not remove shared payload resistance (23).

No clinical study isolates target change from payload, linker, treatment line, and selection. The whole construct should therefore be compared: target, antibody, internalization, linker and release mechanism, active catabolite, payload class, pharmacokinetics, and toxicity. Target-, payload-, or architecture-switching remains a mechanistic consideration rather than a validated rule unless the proposed resistance feature predicts benefit prospectively.

## 7 Toxicity and treatment feasibility

Sequential feasibility depends on whether toxicity is recurrent, cumulative, or agent-specific. Direct evidence quantifying these categories across ADC orders is sparse. Agent-specific liabilities differ: T-DM1 is associated with thrombocytopenia, hepatotoxicity, and neuropathy; SG with neutropenia and diarrhea; T-DXd with nausea, cytopenias, and ILD/pneumonitis; and Dato-DXd with stomatitis, ocular toxicity, and ILD/pneumonitis (2–4, 25). Overlapping toxicities may constrain treatment feasibility but do not demonstrate tumor cross-resistance.

In Huppert et al., 14 of 84 patients developed investigator-reported ILD/pneumonitis during T-DXd; seven received T-DXd as ADC1 and seven as ADC2. The cohort was not designed to compare ILD risk by order and cannot establish cumulative pulmonary toxicity (13). Current T-DXd and Dato-DXd labels require permanent discontinuation for confirmed grade 2 or higher ILD/pneumonitis. Dato-DXd breast cancer studies excluded patients with prior ILD requiring corticosteroids or ongoing ILD, leaving risk after T-DXd-associated ILD uncertain (2, 4). These requirements apply to the implicated agent; available evidence supports neither a class-wide contraindication to every subsequent ADC nor an assumption that a subsequent ILD-liable ADC is safe.

Before ADC2, clinicians should determine whether ADC1 was discontinued because of progression or toxicity and reassess resolution of prior adverse events, pulmonary history, neuropathy, marrow reserve, hepatic and renal function, cardiac status, supportive-care requirements, competing treatment options, and patient priorities. These factors inform treatment feasibility but do not identify the underlying tumor-resistance mechanism.

## 8 Biomarkers and re-biopsy

Re-biopsy at progression can be useful when receptor conversion, histology, or an actionable alteration would change available therapy. It samples one lesion and cannot map spatial heterogeneity; decalcified bone and limited tissue can also impair testing. ASCO/CAP does not define HER2-low as a separate interpretive category, and eligibility should follow validated IHC/ISH methods and the applicable label (4, 26). Re-biopsy is a conditional reassessment tool, not a validated ADC2 selector.

ctDNA can detect genomic alterations, including acquired *TOP1* variants, but cannot directly measure surface-antigen density, spatial distribution, internalization, lysosomal processing, linker cleavage, or bystander effect (23). A negative plasma result does not establish sensitivity to another TOP1 ADC. ZEPHIR-02 was designed to evaluate ^89^Zr-trastuzumab PET/CT after T-DXd as an investigational whole-body HER2 assessment; the study was terminated early because of poor accrual, and the registry establishes design, not clinical utility (27). Tissue, plasma, and imaging access also affect who can enter biomarker-driven trials; evidence development should report sampling feasibility and representation, not only assay performance.

## 9 A subtype-specific clinical framework

**Table 3** organizes decision variables and approved treatment options; it does not rank ADCs or constitute a prospectively validated sequencing algorithm. Decisions should integrate the reason ADC1 was discontinued; subtype and label-defined eligibility; prior exposure; target, linker, payload, and toxicity profiles; CNS disease; disease tempo and distribution; performance status; organ and marrow reserve; prior adverse events; non-ADC alternatives; access; and patient goals. Prospective clinical evidence should outweigh unvalidated mechanistic inference.

**Table 3:** Subtype-specific decision framework after a prior ADC.

Clinical setting	Evidence anchor	Reassessment before ADC2	Decision-relevant interpretation	Inference not supported
HER2-positive advanced/metastatic disease	Current line-specific trials/labels; DESTINY-Breast02 supports T-DXd after T-DM1; DESTINY-Breast12 documents intracranial T-DXd activity (9, 29)	Why ADC1 stopped; current HER2 if result would change therapy; CNS disease; prior T-DM1/T-DXd; ILD, platelets, neuropathy; cardiac, hepatic, marrow reserve	Compare remaining HER2-directed and non-ADC options, including CNS-active regimens; assess target plausibility and complete-construct differences	DESTINY-Breast03 supplies within-patient sequence evidence; payload switching caused benefit; T-DM1 has uniformly low activity after T-DXd
HR-positive, HER2-low/ultralow metastatic disease	Separate prospective indications for T-DXd, SG, and Dato-DXd; retrospective SG/T-DXd order evidence only (12, 13, 31–34)	Endocrine sensitivity; exact HER2/HR eligibility; prior systemic and ADC exposure; CNS disease; ILD, stomatitis/ocular, diarrhea/neutropenia; organ/marrow reserve	Anchor to prospective evidence for the current line; recognize shared TOP1 class; compare non-ADC and biomarker-directed alternatives	Target switching overcomes TOP1 resistance; retrospective PFS2/TTF establishes an order; TROP2 IHC is a validated binary selector
Triple-negative advanced/metastatic disease	Current first-line SG and Dato-DXd indications depend on immunotherapy eligibility; T-DXd requires HER2 eligibility (2–4)	PD-L1/immunotherapy eligibility; actionable germline/somatic biomarkers; HER2 status; CNS disease; prior ADC; pulmonary, marrow, GI, oral/ocular risks	Plan non-ADC and trial options early; if another TOP1 ADC is considered, discuss uncertain benefit and shared class	First-line approval provides sequence-order evidence; response to ADC1 predicts ADC2; absence of a ctDNA alteration proves sensitivity
Any subtype	No completed randomized alternative-order trial	Patient goals; performance status; pace/sites; toxicity attribution; dose intensity; organ/marrow reserve; access; alternatives; trial eligibility	Document whether the strategy is sequential monotherapy, alternation, or combination; prefer a trial when the decision depends on unvalidated biology	Shorter ADC2 duration alone proves cross-resistance; intervening chemotherapy restores sensitivity; toxicity is uniformly cumulative across ADCs

This framework is decision support, not a treatment guideline or validated sequence algorithm. Indications and alternatives must be checked in the relevant jurisdiction at the time of treatment. ADC, antibody–drug conjugate; CNS, central nervous system; HR, hormone receptor; IHC, immunohistochemistry; ILD, interstitial lung disease; SG, sacituzumab govitecan; T-DM1, trastuzumab emtansine; T-DXd, trastuzumab deruxtecan; TNBC, triple-negative breast cancer; TOP1, topoisomerase I.

### 9.1 HER2-positive disease

Prior actual exposure—not a historical sequence—defines the next decision. T-DXd plus pertuzumab is now FDA approved as first-line therapy for unresectable or metastatic HER2-positive breast cancer (28). DESTINY-Breast02 supports T-DXd after T-DM1 but not reverse order or payload-rescue causality (9). CNS disease can materially alter option value: in the noncomparative DESTINY-Breast12 cohort with baseline brain metastases (n = 263), intracranial ORR was 71.7% among the 138 patients with measurable baseline CNS disease, including those with stable and active brain metastases (29). This establishes intracranial activity, not sequence superiority. Tucatinib–trastuzumab–capecitabine is an evidence-based non-ADC CNS-active alternative, but its precise order after contemporary first-line T-DXd exposure is not resolved by HER2CLIMB (30).

### 9.2 HR-positive, HER2-low or HER2-ultralow disease

T-DXd has prospective randomized evidence in HER2-low and HER2-ultralow populations from DESTINY-Breast04 and DESTINY-Breast06, respectively (31, 32). SG and Dato-DXd have separate prospective randomized evidence from TROPiCS-02 and TROPION-Breast01, which enrolled broader HR-positive/HER2-negative populations rather than specifically HER2-low or HER2-ultralow disease (33, 34). These trials evaluated different populations and treatment settings and do not establish the optimal sequence among these ADCs. T-DXd and Dato-DXd deliver DXd, whereas SG releases SN-38; both payloads inhibit TOP1. Retrospective SG/T-DXd cohorts demonstrate residual ADC2 activity but do not establish a preferred order (12, 13). Decisions should incorporate endocrine sensitivity; prior endocrine, targeted, chemotherapy, and ADC exposure; current HER2 eligibility; CNS disease; toxicity; physiologic reserve; and evidence-supported non-ADC or biomarker-directed options. A shared TOP1 payload class informs uncertainty but is not an automatic exclusion criterion.

### 9.3 Triple-negative disease

Earlier-line TROP2 ADC availability enlarges the sequencing problem without resolving it. As of June 24, 2026, SG had first-line US indications as monotherapy when PD-1/PD-L1 therapy was unsuitable and with pembrolizumab for PD-L1 CPS 10 or higher; Dato-DXd was indicated for unresectable or metastatic TNBC when PD-1/PD-L1 therapy was unsuitable (2, 3). T-DXd requires label-defined HER2 eligibility. Immunotherapy eligibility, germline and other actionable biomarkers, CNS disease, toxicity, and non-ADC options remain central. First-line approval is not sequence-order evidence.

## 10 Emerging ADCs and research priorities

Six registry studies address distinct post-ADC questions. TRADE-DXd, SERIES, and STORM study protocol-defined sequential strategies; ALTER studies alternation; SATEEN studies SG plus trastuzumab after T-DXd; and ZEPHIR-02 evaluated molecular imaging before T-DM1 until its early termination for poor accrual (27, 35–39). A protocol containing multiple ADCs is not necessarily a randomized order comparison, and registry records support design and status—not efficacy.

Priority studies should compare order in clinically comparable populations; preserve PFS, rwPFS, TTF, ORR, duration of response, and OS as distinct endpoints; capture intervening therapy, dose intensity, sequential toxicity, patient-reported outcomes, and organ reserve; and prespecify CNS eligibility and intracranial endpoints. Longitudinal tissue, ctDNA, and justified whole-body target imaging should test, rather than assume, target or payload mechanisms. Bispecific, biparatopic, site-specific, dual-payload, and immune-stimulatory constructs remain investigational until studied after defined prior ADC exposure. Research should also address tissue-testing access, demographic representation, comorbidity, and community generalizability.

## 11 Discussion

The evidence supports residual ADC2 activity in a subset, but not a universal order or a single mechanism explaining diminishing benefit. Human longitudinal studies show that target and payload resistance can occur and coexist; their selected samples do not establish population frequency or predictive performance. Processing failure, efflux, and rescue through construct switching remain mainly preclinical. The clinically useful synthesis is therefore a disciplined comparison of the whole construct within the patient’s evolving disease and tolerance, while keeping mechanistic switching explicitly unvalidated.

## 12 Limitations

This structured narrative review did not use duplicate systematic screening, a formal exclusion log, quantitative pooling, or a risk-of-bias instrument. The clinical evidence is predominantly retrospective, heterogeneous, and vulnerable to selection, later-line, treatment-exposure, and endpoint biases. Translational studies use small or selected tissue and plasma cohorts; models may not reproduce human heterogeneity, immunity, exposure, or treatment history. Regulatory and registry status is date-specific. The framework cannot establish causal order or validated biomarker-guided selection.

## 13 Conclusions

A second ADC can retain activity, but shorter ADC2 outcomes do not by themselves prove cross-resistance, and no universally optimal order is established. Target and payload changes may matter, yet switching one construct component cannot be assumed to bypass failure at another level. Decisions should be anchored to subtype-specific prospective evidence and current indications, then individualized by the reason ADC1 stopped, target eligibility, CNS disease, toxicity, reserve, alternatives, access, and patient goals. Prospective order studies with standardized endpoints, longitudinal biospecimens, CNS assessment, and sequential safety and quality-of-life measurement are required before mechanistic plausibility can support a clinical algorithm.
